# Immunogenicity and Safety of 3 Formulations of a Respiratory Syncytial Virus Candidate Vaccine in Nonpregnant Women: A Phase 2, Randomized Trial

**DOI:** 10.1093/infdis/jiz395

**Published:** 2019-08-16

**Authors:** Tino F Schwarz, Roderick A McPhee, Odile Launay, Geert Leroux-Roels, Jaak Talli, Marta Picciolato, Feng Gao, Rongman Cai, Thi Lien-Anh Nguyen, Ilse Dieussaert, Jacqueline M Miller, Alexander C Schmidt

**Affiliations:** 1 Institute of Laboratory Medicine and Vaccination Centre, Klinikum Würzburg Mitte, Würzburg, Germany; 2 GlaxoSmithKline (GSK), Rockville, Maryland; 3 Université de Paris, Inserm, clinical investigation center 1417, Assistance Publique–Hôpitaux de Paris, Hôpital Cochin, Paris, France; 4 Center for Vaccinology, Ghent University and University Hospital, Belgium; 5 Ravi-ja Uuringukeskus Innomedica OÜ, Tallinn, Estonia; 6 GSK, Rixensart; 7 GSK, Wavre, Belgium

**Keywords:** respiratory syncytial virus, randomized trial, nonpregnant women, safety, neutralizing antibodies, palivizumab competing antibody, maternal immunization

## Abstract

**Background:**

Respiratory syncytial virus (RSV) is a common cause of respiratory tract illness and hospitalization in neonates and infants. RSV vaccination during pregnancy may protect offspring in their first months of life.

**Methods:**

This randomized, observer-blind, multicenter, phase 2 study evaluated the immunogenicity and safety of an RSV candidate vaccine in healthy nonpregnant women aged 18–45 years. Four hundred participants were randomized (1:1:1:1) to receive a single intramuscular dose of vaccine containing 30 µg, 60 µg, or 120 µg of RSV fusion protein engineered to preferentially maintain a prefusion conformation (RSV-PreF vaccine) or placebo.

**Results:**

Thirty days postvaccination, RSV-A neutralizing antibody geometric mean titers (GMTs) increased 3.75-, 4.42- and 4.36-fold; RSV-B neutralizing antibody GMTs 2.36-, 2.54- and 2.76-fold; and palivizumab competing antibody (PCA) concentrations 11.69-, 14.38- and 14.24-fold compared with baseline levels in the 30 µg, 60 µg, and 120 µg RSV-PreF groups, respectively. Antibody titers and PCA concentrations at day 30 were significantly higher with the 120 µg compared to the 30 µg RSV-PreF vaccine. All RSV-PreF vaccine formulations and the placebo had similar reactogenicity profiles. No serious adverse events were considered to be related to the RSV-PreF vaccine.

**Conclusions:**

The 3 formulations of the investigational RSV-PreF vaccine were well-tolerated and induced RSV-A and RSV-B neutralizing antibodies and PCAs in healthy, nonpregnant women.

**Clinical Trials Registration:**

NCT02956837.

## Teaser

Three formulations of an investigational maternal respiratory syncytial virus (RSV) vaccine containing 30, 60, or 120 µg of antigen were well-tolerated and elicited a rise in RSV-A and RSV-B neutralizing antibody titers as well as palivizumab-competing antibody titers in healthy nonpregnant women.

Respiratory syncytial virus (RSV), a member of the Pneumoviridae family, is a highly contagious human pathogen. RSV causes respiratory tract infections in all age groups with hospitalizations primarily reported in infants and the elderly, representing a major global health and economic burden [1–3]. Most children have been infected with RSV by the age of 2 years [4, 5], with severe lower respiratory tract illness being most common in infants <6 months of age [5–7].

Although a monoclonal antibody (palivizumab [Synagis], MedImmune) is indicated for the prevention of severe RSV lower respiratory tract infections (LRTIs) in high-risk infants [1, 8, 9], no vaccine is available and treatment of RSV disease remains largely symptomatic [3]. The development of an RSV vaccine for neonates and infants has been challenging, mainly because natural infection does not induce sterile immunity and RSV is able to temporarily evade innate immune responses [10].

Passive protection of young infants may be achieved through vaccination of pregnant women, as currently recommended for inactivated influenza and pertussis vaccines in a large number of countries worldwide [11–15], and for tetanus vaccination in low- and middle-income countries [16]. Since almost all adolescents and adults have preexisting immunity against RSV, the administration of a RSV vaccine is likely to boost maternal antibody titers and protect infants by placental antibody transfer [15, 17].

The RSV fusion protein (RSV-F) is a major surface glycoprotein that mediates the fusion of the viral envelope with the target cell membrane and enables virus entry into respiratory epithelial cells [18]. During membrane fusion, RSV-F undergoes a conformational change from a prefusion (PreF) to a postfusion state. The PreF conformation exposes additional epitopes for highly potent neutralizing antibodies [19–22]. The vaccine antigen (RSV-PreF) used in our study is an RSV-F protein, engineered to preferentially maintain a prefusion conformation, which has been previously evaluated in other clinical trials at antigen dose levels up to 60 µg [23, 24]. A phase 1 study in men aged 18–44 years showed that aluminum-adjuvanted and unadjuvanted formulations of the RSV-PreF vaccine containing 10 µg, 30 µg, or 60 µg of PreF protein were able to boost neutralizing antibody titers [23], which are essential for protection against RSV-associated illness [19, 25]. The formulations containing higher doses of PreF antigen were more immunogenic, but the aluminum adjuvant did not significantly enhance immune responses [23]. Beran et al described 2 phase 2 trials using that same antigen in nonpregnant women [24]. In the first trial, participants received a single dose of unadjuvanted RSV-PreF vaccine containing either 30 µg or 60 µg of PreF protein, the aluminum-adjuvanted RSV-PreF containing 60 µg of PreF protein, or an adult formulation of combined tetanus toxoid–diphtheria toxoid–acellular pertussis vaccine. Results showed no beneficial effect of the aluminum adjuvant in terms of neutralizing antibody titers and higher rates of injection site pain and general adverse events (AEs), including fatigue and headache with the aluminum-adjuvanted formulation. These results led to the selection of unadjuvanted RSV-PreF formulation for the second phase 2 trial described by Beran et al, which confirmed that the same unadjuvanted RSV-PreF vaccine containing 60 μg of PreF protein was well-tolerated in 18- to 45-year-old nonpregnant women [24].

Here, we present the results of a more recent phase 2 study evaluating the safety, reactogenicity, and immunogenicity of 3 formulations of the above-mentioned unadjuvanted RSV-PreF vaccine, containing 30 µg, 60 µg, and, for the first time, 120 µg of PreF protein in nonpregnant women of childbearing age. The formulation containing 120 µg of PreF was studied to further define a dose-response curve and select a safe and effective RSV-PreF vaccine formulation.

## METHODS

### Study Design and Vaccines

**Figure 1.**
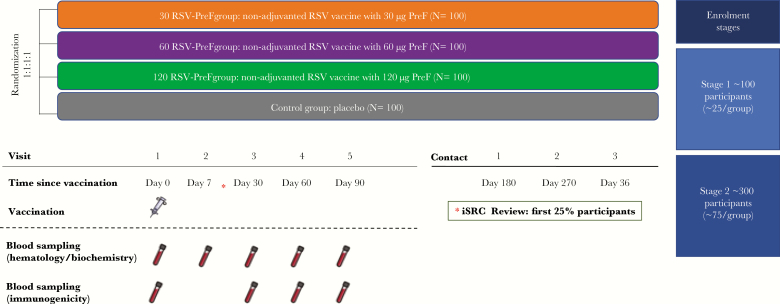
Study design and procedures. Enrollment stages reflect planned enrollment figures. Abbreviations: 30 RSV-PreF/60 RSV-PreF/120 RSV-PreF, group of women who received 1 dose of the unadjuvanted respiratory syncytial virus (RSV) vaccine containing 30, 60, or 120 μg of RSV prefusion F protein; Control, group of women who received 1 dose of placebo; iSRC, internal safety review committee.

This phase 2, observer-blind, randomized controlled study was conducted in 8 centers in Belgium, Estonia, France, and Germany between November 2016 and February 2018. Healthy, nonpregnant women were randomized into 4 parallel groups (1:1:1:1) to receive 1 dose of unadjuvanted RSV-PreF vaccine containing 30 μg (30 RSV-PreF group), 60 μg (60 RSV-PreF group), or 120 µg (120 RSV-PreF group) of PreF protein, or 1 dose of placebo (control group) (Figure 1). Randomization was done using a centralized randomization system. The randomization algorithm used a minimization procedure accounting for age and center.

As this was the first clinical trial evaluating the RSV-PreF vaccine formulation containing 120 µg of PreF protein, a 2-step staggered enrollment with an unblinded review of safety data by a GSK internal safety review committee (iSRC) was performed. In the first step, 25% of participants were enrolled. The initiation of the second enrollment step (75% of participants) was dependent on a positive outcome of the unblinded review of safety and reactogenicity data collected up to at least 7 days postvaccination by the GSK iSRC.

This study was conducted in an observer-blind manner up to 90 days postvaccination (day 90). After day 90, the statisticians were unblinded and the study was conducted in a single-blind manner.

All vaccine formulations were presented as freeze-dried antigen in monodose vials and were reconstituted using normal saline. Women in the control group received a saline-based placebo. The study vaccines and placebo were administered intramuscularly in a volume of 0.5 mL in the deltoid region of the arm.

This study was conducted in accordance with the International Conference on Harmonization Guidelines for Good Clinical Practice, all applicable privacy requirements, and the guiding principles of the Declaration of Helsinki. Institutional review boards/independent ethics committees at each institution reviewed and approved the study protocol, amendments, and informed consent forms. Written informed consent was obtained from each participant prior to the performance of any study-specific procedures. The study is registered at ClinicalTrials.gov (NCT02956837). The protocol is available at http://www.gsk-clinicalstudyregister.com (ID204812); it includes a list of 5 secondary endpoints, of which 4 are reported in this manuscript. Anonymized individual participant data and study documents can be requested for further research at https://www.clinicalstudydatarequest.com.

### Study Participants

Participants were healthy, nonpregnant women aged 18–45 years who had given written informed consent. Women of childbearing potential practiced adequate contraception for 30 days prior to vaccination, had a negative pregnancy test on the day of vaccination, and agreed to continue adequate contraception up to day 90. The full list of exclusion criteria is given in Supplementary Data 1.

### Study Objectives

The primary objective was to rank the 3 investigational RSV-PreF vaccine formulations based on the calculation of a desirability index using immunogenicity, safety, and reactogenicity data. The primary endpoints for safety and reactogenicity were occurrence within 7 days postvaccination of (*i*) any grade 2/3 general AE, (*ii*) vaccine-related serious AE (SAE), and (*iii*) grade 2/3 fever (temperature >38.5°C). Neutralizing antibody titers against RSV-A and palivizumab competing antibody (PCA) concentrations at day 30 postvaccination adjusted for prevaccination titers were selected as primary immunogenicity endpoints for the desirability index (Supplementary Data 2). This is described in more detail in the Statistical Analysis section.

The secondary objectives were to evaluate the reactogenicity and safety of the RSV-PreF vaccine formulations up to 360 days postvaccination (day 360), their immunogenicity up to day 90, and the incidence of medically attended RSV-associated respiratory tract infections up to day 360.

### Safety Assessment

Details of the safety assessment are provided in Supplementary Data 3.

### Immunogenicity Assessment

Blood samples for immunogenicity assessments were collected on days 0, 30, 60, and 90 and analyzed as described in Supplementary Data 4.

Neutralizing antibody titers against RSV-A and RSV-B and PCA concentrations were measured up to day 90, and RSV-specific total immunoglobulin (IgG) antibody titers and IgG1 antibody titers up to day 30. For RSV-A, a titer of ≥8 was defined as seropositive. For RSV-B, a titer of ≥6 was defined as seropositive. The lower limit of quantitation for the PCA assay was 9.60 µg/mL.

### Statistical Analysis

Details of the statistical analysis are provided in Supplementary Data 5.

As mentioned in the protocol, the primary objective of the study was to rank the RSV-PreF vaccine formulations by calculating a desirability index using multiple endpoints. The multicriteria decision-making approach was used to identify a function and create a desirability index for each endpoint (“0” being considered as not desirable and “1” as the most desirable). An overall desirability index for each formulation was calculated by computing a weighted geometric mean of the endpoint indices [26]. However, the initial statistical analysis plan was amended prior to data unblinding and analysis to include conventional statistics as the derived endpoints computed and considered in the desirability analysis were felt to potentially introduce biases, for example, by assigning equal weights to grade 2/3 AEs and SAEs. The desirability index approach was used as a descriptive tool only to support the formulation ranking.

The sample size was determined to allow reliable ranking of the RSV-PreF formulations based on the desirability index. Simulations indicated that a study with 95 evaluable participants per group would have an 80% chance to select a superior formulation.

## RESULTS

### Study Population

**Figure 2.**
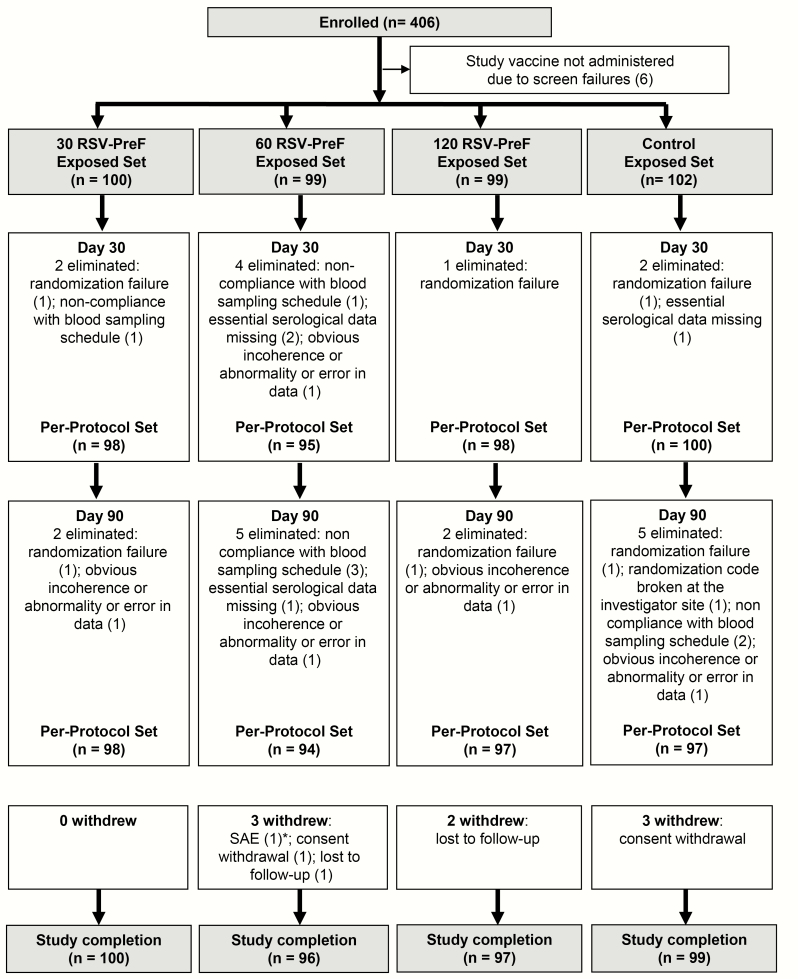
Flow of participants. *One participant was withdrawn due to a serious adverse event after contact 2 (day 270). Abbreviations: 30 RSV-PreF/60 RSV-PreF/120 RSV-PreF, group of women who received 1 dose of the unadjuvanted respiratory syncytial virus (RSV) vaccine containing 30, 60, or 120 μg of RSV prefusion F protein; Control, group of women who received 1 dose of placebo; SAE, serious adverse event.

Of 406 women enrolled in this study, 400 were vaccinated (100 in the 30 RSV-PreF group, 99 in the 60 RSV-PreF group, 99 in the 120 RSV-PreF group, and 102 in the control group) and 392 completed the study (Figure 2). The per-protocol sets included 391 women on day 30 and 386 on day 90. The demographic characteristics of participants were well-balanced between groups (Table 1).

**Table 1.** Demographic Characteristics of the Study Participants at Enrollment (Exposed Set)

Characteristic	30 RSV-PreF (n = 100)	60 RSV-PreF (n = 99)	120 RSV-PreF (n = 99)	Control (n = 102)
Age at vaccination, y				
Mean (SD)	30.2 (6.7)	29.1 (7.2)	29.6 (7.1)	29.9 (6.9)
Range	18–45	18–44	18–44	20–44
Geographic ancestry, No. (%)				
Asian^a^	1 (1.0)	1 (1.0)	1 (1.0)	1 (1.0)
African/African American	1 (1.0)	0 (0.0)	2 (2.0)	1 (1.0)
White, Caucasian/European	96 (96.0)	97 (98.0)	93 (93.9)	98 (96.1)
White, Arabic/North African	0 (0.0)	1 (1.0)	2 (2.0)	1 (1.0)
Other	2 (2.0)	0 (0.0)	1 (1.0)	1 (1.0)

Case groups refer to groups of women who received 1 dose of the unadjuvanted RSV vaccine containing 30, 60, or 120 μg of RSV PreF. Control refers to the group of women who received 1 dose of the placebo.

Abbreviations: PreF, prefusion F protein; RSV, respiratory syncytial virus; SD, standard deviation.

^a^Asian refers to participants of Central/South Asian, East Asian, Japanese, or Southeast Asian heritage.

### Ranking of RSV-PreF Vaccine Formulations

**Figure 3.**
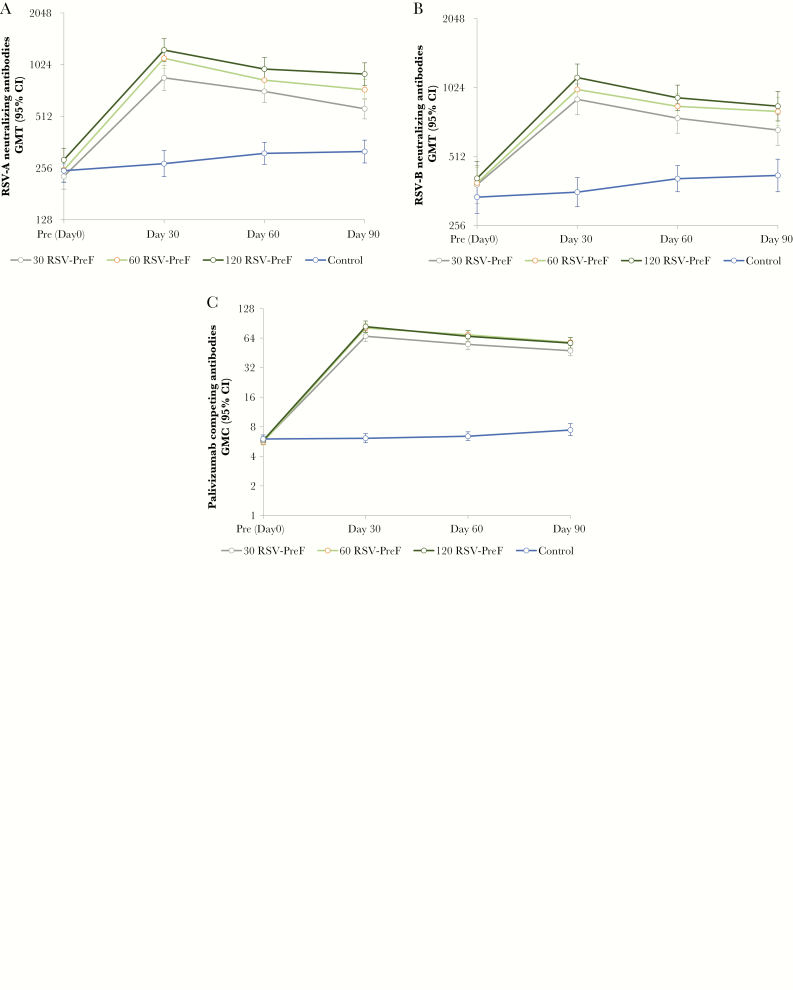
Geometric mean respiratory syncytial virus–A neutralizing antibody titers (*A*), geometric mean respiratory syncytial virus–B neutralizing antibody titers (*B*), and geometric mean palivizumab-competing antibody concentrations (*C*) until day 90 (per-protocol set). Error bars represent 95% confidence intervals. The raw data for the 60 μg and 120 μg groups are so similar as to be indistinguishable in Figure 3C. Abbreviations: 30 RSV-PreF/60 RSV-PreF/120 RSV-PreF, group of women who received 1 dose of the unadjuvanted RSV vaccine containing 30, 60, or 120 μg of RSV prefusion F protein; CI, confidence interval; Control, group of women who received 1 dose of placebo; GMC, geometric mean concentration; GMT, geometric mean titer; RSV, respiratory syncytial virus.

Although RSV-A and RSV-B neutralizing titers at day 30 increased with antigen dose (Figures 3A and 3B), differences in titer were only statistically significant for the comparison of 120 RSV-PreF vs 30 RSV-PreF, but not for the comparison of 120 RSV-PreF vs 60 RSV-PreF or 60 RSV-PreF vs 30 RSV-PreF. The same was true for PCA titers; that is, the difference in PCA GMCs was only statistically significant for the comparison of 120 RSV-PreF vs 30 RSV-PreF. The safety data suggested that all vaccine formulations were well-tolerated, with no significant differences between groups with regard to the safety endpoints.

Because safety and reactogenicity did not differ much between treatment groups, the desirability index—combining immunogenicity and safety—did not add much over a comparison of immunogenicity. The overall desirability indices, which were 0.19, 0.24, and 0.27 for the 30, 60, and 120 RSV-PreF groups, respectively, and 0.05 for the control group, were only used descriptively and not to select a superior formulation of the RSV-PreF vaccine as clinical development of this antigen was discontinued (Supplementary Table 1).

### Immunogenicity

In the per-protocol set, all participants were seropositive for RSV-A and RSV-B neutralizing antibodies at prevaccination. By day 30, RSV-A neutralizing antibody GMTs had increased 3.75-fold, 4.42-fold, and 4.36-fold in the 30, 60, and 120 RSV-PreF groups, respectively. At day 30, RSV-A neutralizing antibody GMTs ranged between 858.2 and 1245.5 in active treatment groups, with the highest GMTs observed in the 120 RSV-PreF group. RSV-A neutralizing antibody GMTs remained higher than baseline levels at day 90 in the 30 (2.49-fold), 60 (2.94-fold), and 120 (3.12-fold) RSV-PreF groups (Figure 3A).

RSV-B neutralizing antibody GMTs increased between baseline and day 30 in the 30 (2.36-fold), 60 (2.54-fold), and 120 (2.76-fold) RSV-PreF groups. At day 30, RSV-B neutralizing antibody GMTs ranged between 909.1 and 1131.7 in active treatment groups, with the highest GMTs observed in the 120 RSV-PreF group. RSV-B neutralizing antibody GMTs remained above baseline levels at day 90 in the 30 (1.75-fold), 60 (2.09-fold), and 120 (2.01-fold) RSV-PreF groups (Figure 3B).

At prevaccination, 16.0%–22.0% of participants had detectable PCA concentrations, although GMCs were close to the assay cutoff. PCA GMCs increased between baseline and day 30 in the 30 (11.69-fold), 60 (14.38-fold), and 120 (14.24-fold) RSV-PreF groups, but not in the control group (1.02-fold), and remained above baseline values at day 90 in the investigational groups (8.25- to 10.16-fold) (Figure 3C).

Exploratory comparisons for immunogenicity between the 120 and 30 RSV-PreF groups at day 30 showed a ratio of 1.31 (95% confidence interval [CI], 1.04–1.64) for RSV-A neutralizing antibodies, 1.20 (95% CI, 1.01–1.43) for RSV-B neutralizing antibodies, and 1.23 (95% CI, 1.00–1.51) for PCA concentrations, indicating that the rise in immune responses with increasing dose levels was statistically significant between the highest and the lowest dose levels only. Comparisons between neighboring dose levels (120 RSV-PreF vs 60 RSV-PreF and 60 RSV-PreF vs 30 RSV-PreF) did not detect statistically significant differences. Between baseline and day 30, RSV F-specific total IgG and IgG1 antibody titers increased in the 30 (19.64- and 13.44-fold), 60 (24.56- and 16.19-fold), and 120 (31.60- and 18.64-fold) RSV-PreF groups.

Exploratory comparisons showed that the ratios between the fold increases in RSV-F–specific total IgG antibody titers and in RSV-A neutralizing antibody titers between baseline and day 30 were 5.89 (95% CI, 4.57–7.58) in the 30 RSV-PreF group, 5.86 (95% CI, 4.65–7.39) in the 60 RSV-PreF group, 6.74 (95% CI, 5.35–8.50) in the 120 RSV-PreF group, and 1.06 (95% CI, .92–1.22) in the control group.

### Safety

Vaccine-related SAEs were not reported in any active treatment arm. Two women reported fever >38.5°C within 7 days postvaccination. The frequency of grade 2/3 AEs (solicited and unsolicited) during the 7-day postvaccination period were 34.0% (95% CI, 24.8%–44.2%) in the 30 RSV-PreF group, 35.4% (95% CI, 26.0%–45.6%) in the 60 RSV-PreF group, 28.3% (95% CI, 19.7%–38.2%) in the 120 RSV-PreF group, and 26.5% (95% CI, 18.2%–36.1%) in the control group. Exploratory analyses did not detect any differences between groups for the above endpoints (Supplementary Table 2).

**Figure 4.**
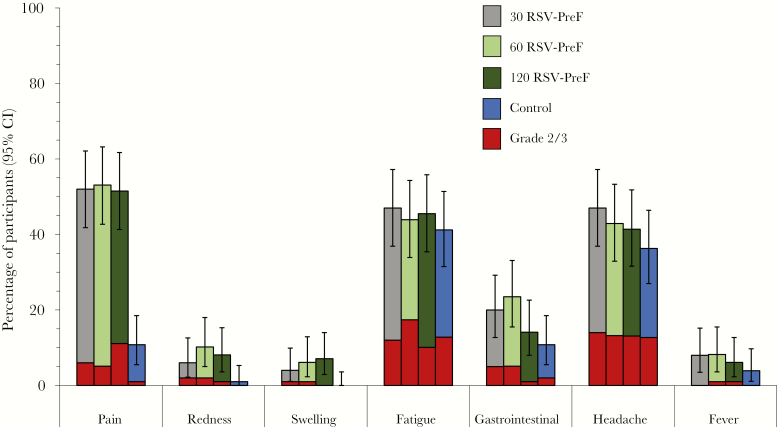
Solicited injection site and general adverse events reported within 7 days after vaccination (exposed set). Error bars represent 95% confidence intervals. Abbreviations: 30 RSV-PreF/60 RSV-PreF/120 RSV-PreF, group of women who received 1 dose of the unadjuvanted respiratory syncytial virus (RSV) vaccine containing 30, 60, or 120 μg of RSV prefusion F protein; AEs, adverse events; CI, confidence interval; Control, group of women who received 1 dose of placebo.

Up to day 7, the most frequently reported solicited local AE was mild to moderate injection site pain, experienced by approximately half of women in the investigational groups and 10.8% of women in the control group (Figure 4). The most frequently reported solicited general AEs were fatigue (41.2%–47.0%) and headache (36.3%–47.0%). Gastrointestinal AEs and fever (temperature ≥37.5°C) were reported by ≤23.5% and ≤8.2% of participants, respectively. One participant reported grade 3 fever on day 0 after administration of the vaccine (temperature >39.5°C; 60 RSV-PreF group), which was not considered as vaccine-related. No increased rates of grade 3 solicited local and general AEs were reported with increasing dose levels (Figure 4).

Up to day 7, solicited and unsolicited AEs were reported by 84.0% (30 RSV-PreF group), 84.8% (60 RSV-PreF group), 78.8% (120 RSV-PreF group), and 63.7% (control group) of women. Up to day 30, the most frequently reported solicited and unsolicited AEs were headache, rhinitis, oropharyngeal pain, upper respiratory tract infection, cough, and nasopharyngitis.

Ten SAEs, none considered to be vaccine-related by the investigator, were reported by 8 participants up to study end: transient grade 3 liver enzyme elevations ascribed to biliary colic on day 33 in the 30 RSV-PreF group; soft tissue infection on day 62, spontaneous abortion on day 107, and lung adenocarcinoma and pancreatic carcinoma on day 319 in the 60 RSV-PreF group; and *Campylobacter* gastroenteritis on day 79 and hemiparesis on day 248 in the 120 RSV-PreF group. In the control group, 1 participant developed a peritonsillar abscess on day 63, and 1 participant had a spontaneous pneumothorax on day 20 and rheumatoid arthritis on day 103. No fatalities were reported.

Medically attended respiratory tract illnesses were reported by 21.0% (95% CI, 13.5%–30.3%) of participants in the 30 RSV-PreF group, 11.1% (95% CI, 5.7%–19.0%) in the 60 RSV-PreF group, 10.1% (95% CI, 5.0%–17.8%) in the 120 RSV-PreF group, and 8.8% (95% CI, 4.1%–16.1%) in the control group. A total of 13 pregnancies were reported in 13 participants during the study. Outcomes at study end were recorded as live births in 5 participants, spontaneous abortion in 1 participant (60 RSV-PreF group), and ongoing pregnancies in 7 participants. No apparent congenital anomalies were reported.

Clinically significant changes in hematologic and biochemical parameters were not noted between day 7 and day 90 in the 60 and 120 RSV-PreF groups. In the 30 RSV-PreF group, 1 participant, with normal liver biochemistries at day 7, developed a transient grade 3 increase in alanine aminotransferase and a transient grade 4 increase in aspartate aminotransferase at day 30. Both laboratory values returned to normal by day 60. This participant was diagnosed with severe biliary colic, which was reported as an SAE, resolved by day 60, and was not considered related to vaccination.

## DISCUSSION

This phase 2 study showed that 1 dose of investigational RSV-PreF vaccine, containing either 30 μg, 60 μg, or 120 µg of RSV-PreF protein, boosted preexisting immune responses to RSV in healthy women of childbearing age. All dose levels evaluated in this study were well tolerated. Our results are in line with previous phase 1/2 studies of this antigen conducted in healthy men and nonpregnant women, but this was the first study to explore the 120 µg dose level of RSV-PreF [23, 24]. In the protocol, a desirability index was defined to select a superior vaccine formulation based on safety, reactogenicity, and immunogenicity data. However, as safety and reactogenicity profiles were comparable between groups, the desirability indices simply reflected the ranking of the immunogenicity results and were therefore not useful to select a superior formulation of the RSV-PreF vaccine.

As expected in an adult population, all women were seropositive for RSV-A and RSV-B neutralizing antibodies at prevaccination. Neutralizing antibody GMTs against RSV-A and RSV-B increased approximately 4- to 5-fold between baseline and 30 days postvaccination. The fold-rise in neutralizing antibody GMTs was similar in either the 60 µg or the 120 µg treatment group, suggesting that these antigen dose levels are near the upper end of the dose-response curve. Although neutralizing antibody titers waned at later timepoints, they remained 2- to 4-fold above baseline at 90 days postvaccination, which is in line with previous studies [23, 24] and could potentially be sufficient to protect infants for the first several months of life [27, 28]. Previous antigenic characterization [29] as well as potency evaluation [30] studies have suggested that higher fold-rises in neutralizing antibody titers can potentially be achieved with optimized antigens. In this context, several prefusion F antigens are being evaluated in preclinical and clinical studies [31]. A recent phase 3 efficacy study on an RSV-F nanoparticle vaccine, one of the RSV maternal vaccines in advanced clinical development, did not meet its primary objective of prevention of medically significant RSV LRTIs during the first 90 days of life (39% efficacy [97.5% CI, –1% to 64%]) [32–34]. Yet, statistically significant efficacy was demonstrated against the secondary objective, that is, RSV LRTI hospitalization during the first 90 days of life (44% efficacy [95% CI, 20%–62%]).

At prevaccination, only a small proportion of women had quantifiable concentrations of PCA, that is, antibodies recognizing the same site as palivizumab, the monoclonal antibody with demonstrated efficacy in preventing RSV lower respiratory tract illness in high-risk infants [10]. In a previous phase 2 trial, quantifiable PCA concentrations were detected in most participants at prevaccination [24]. However, in this previous study, PCA concentrations were close to the assay cutoff and PCA GMCs were comparable to those measured in our study. One possible explanation was that the palivizumab-binding epitope on the PreF antigen differs from the epitope displayed on the surface of the virion, so that polyclonal antibodies induced by natural infection do not efficiently displace palivizumab from that epitope on the PreF vaccine antigen used to coat the enzyme-linked immunosorbent assay (ELISA) plates in this assay. In line with the previous phase 1 and 2 studies, robust increases in PCA GMCs were induced by the different formulations of the RSV-PreF vaccine [23, 24]. Similarly, levels of RSV-F–specific total IgG and IgG1, a subclass of antibodies efficiently transferred across the placenta [35], also increased between baseline and 30 days postvaccination.

The rises in RSV-A neutralizing antibody GMTs (3.75- to 4.36-fold) relative to rises in total IgG (19.64- to 31.60-fold) and PCA titers (11.69- to 14.38-fold) were consistent with those observed in previous trials with this vaccine antigen [24]. While this comparison of fold-rises using different assays needs to be interpreted with caution, it may be used to evaluate different RSV-F protein antigens, as long as the same ELISA and neutralization assays are used for comparison.

Although we did not identify any safety concern in this study or prior studies of this vaccine antigen, the development of the candidate vaccine described here was discontinued prior to the initiation of trials in pregnant women due to instability of the RSV-PreF antigen during the manufacturing process. Clinical development is currently ongoing with a modified RSV-PreF antigen with the expectation to proceed into studies with pregnant women in the near future.

Our study was limited by its observer-blind design due to differences in study vaccine and placebo appearance, which precluded a double-blind design, and by the numerous exploratory analyses, which should be interpreted cautiously. A further limitation was the lack of correlates of protection for the assays used in this study.

In summary, this study showed that the 3 formulations of the investigational RSV-PreF vaccine containing up to 120 µg of RSV-PreF protein were well-tolerated and boosted preexisting immune responses. The rise in antibody titers with increasing dose levels was less than linear and suggests that the benefit of increasing dose levels of this antigen beyond 120 µg may be limited. Clinical development of this antigen has been discontinued.

## Supplementary Data

Supplementary materials are available at *The Journal of Infectious Diseases* online. Consisting of data provided by the authors to benefit the reader, the posted materials are not copyedited and are the sole responsibility of the authors, so questions or comments should be addressed to the corresponding author.

jiz395_suppl_Supplementary_Materials

## Notes

***Acknowledgments.*** The authors thank the participants who took part in the study as well as the study teams who contributed to the study. The authors are grateful to the study investigators (Violaine Corbin, Irmgard Marten, Airi Poder) and the GSK study team (Ouzama Henry, Koen Maleux, and Andrea Sutherland) for their contribution to the study. The authors also acknowledge XPE Pharma & Science c/o GSK: Claire Verbelen provided medical writing services and Quentin Deraedt coordinated the publication. Synagis is a registered trademark of the AstraZeneca group of companies.

***Author contributions.*** A. C. S., F. G., I. D., J. M. M., M. P., O. L., R. C., and T. L.-A. N. were involved in the conception or design of the study. A. C. S., F. G., G. L.-R., I. D., J. M. M., J. T., O. L., R. C., and T. F. S. participated in data collection or generation, or performed the study. A. C. S., F. G., G. L.-R., I. D., J. M. M., R. A. M., R. C., T. F. S., and T. L.-A. N. were involved in data analysis or interpretation. All authors have drafted or reviewed the manuscript, they have all approved the final version of the manuscript as submitted, and all accept full accountability for the content.

***Financial support.*** This work and related publication were sponsored by GlaxoSmithKline Biologicals SA.

***Potential conflicts of interest.*** G. L.-R.’s institution received compensation from the GSK group of companies for study conduct. O. L. reports grants for study conduct from Assistance Publique–Hôpitaux de Paris and personal fees from Sanofi Pasteur, Pfizer, and Janssen. T. F. S. reports personal fees from the GSK group of companies, Pfizer, and Sanofi Pasteur. F. G., I. D., J. M. M., M. P., R. A. M., and T. L.-A. N. are employed by the GSK group of companies and hold shares in the GSK group of companies. A. C. S. and R. C. were employed by the GSK group of companies during the study conduct, and A. C. S. holds GSK shares. J. T. has nothing to disclose.
