# Supplementary material for: Immunogenicity and Safety of 3 Formulations of a Respiratory Syncytial Virus Candidate Vaccine in Nonpregnant Women: A Phase 2, Randomized Trial
Source: J Infect Dis. 2019 Aug 16;220(11):1816–25. doi: 10.1093/infdis/jiz395 (PMC6898794; doi:10.1093/infdis/jiz395)
Supplement: jiz395_suppl_Supplementary_Materials [file jiz395_suppl_supplementary_materials.docx]

**SUPPLEMENTARY MATERIALS**

**Supplementary data 1. List of exclusion criteria**

Potential study participants presenting with one or more of the following criteria were excluded from enrolment in this study:

- Use of any investigational/non-registered drug or vaccine other than the study vaccines within 30 days prior to study vaccination (Day -29 to Day 0), or planned use during the study period.
- Participation in the active phase of another clinical study at any time during the study period, in which the participant has been/will be exposed to an investigational/non-investigational vaccine, pharmaceutical product or device.
- More than 14 days of administration of immunosuppressants or other immune-modifying drugs, administration of long-acting immune-modifying drugs within 6 months prior to study vaccination, or planned administration until 90 days post-vaccination. This corresponds to prednisone ≥ 10 mg/day, or equivalent for corticosteroids (inhaled and topical steroids are not excluded).
- Administration of immunoglobulins and/or any blood products during the 3-month period prior to study vaccination, or planned administration until 90 days post-vaccination.
- Administration of a vaccine not foreseen by the study protocol within the 30-day period prior to and after study vaccination, except for any licensed influenza vaccine which may be administered ≥ 15 days before or after study vaccination.
- Previous experimental vaccination against respiratory syncytial virus.
- History of any neurological disorder or seizure (except for participants with a history of febrile convulsion)
- Family history of congenital or hereditary immunodeficiency.
- Any confirmed or suspected immunosuppressive or immunodeficient condition, based on medical history and physical examination (no laboratory testing required).
- History of or current autoimmune condition (based on the opinion of the investigator and/or specific available diagnostic data).
- Acute or chronic, clinically significant pulmonary, cardiovascular, hepatic or renal functional abnormality as determined by physical examination and/or medical history (including any previously performed laboratory tests).
- Lymphoproliferative disorder or malignancy within previous 5 years (excluding effectively treated non-melanotic skin cancer).
- History of any reaction or hypersensitivity likely to be exacerbated by any component of the study vaccine.
- Hypersensitivity to latex.
- Any medical condition that would make intramuscular injection unsafe according to the investigator’s judgment.
- Current alcohol and/or drug abuse
- Acute disease and/or fever at enrolment: Fever is defined as temperature ≥37.5°C for oral, axillary or tympanic route, or ≥ 38.0°C for rectal route; participants with a minor illness (such as mild diarrhea, mild upper respiratory infection) without fever may be enrolled at the discretion of the investigator; for participants with acute disease and/or fever at the time of enrolment, visit 1 will be rescheduled within the allowed recruitment period.
- Body mass index > 40 kg/m².
- Pregnant or lactating female.
- Planned move to a location that will prohibit the participation in the clinical trial until study conclusion.
- Any other condition that the investigator judges may interfere with study procedures (such as drawing blood) or findings (such as the immune response).

**Supplementary data 2. Desirability index approach**

- **DERIVED ENDPOINTS**

The following endpoints were computed and taken into account in the desirability analysis:

1. Incidence rate of any Grade 2 and any Grade 3 general AE (solicited and unsolicited) and any vaccine-related SAE during the 7-day follow-up period after vaccination for each investigational RSV vaccine formulation.
2. Incidence rate of Grade 2 and Grade 3 fever during the 7-day follow-up period after vaccination for each investigational RSV vaccine formulation.
3. Geometric mean of neutralising antibody titres against RSV-A at Day 30 adjusted for pre-vaccination titres.
4. Geometric mean of PCA concentrations at Day 30 adjusted for pre-vaccination titres.

- **STATISTICAL COMPUTATION**

**Reactogenicity**

A logistic regression model has been fitted on each reactogenicity endpoint (any Grade 2/3 general AE and any related SAE, Grade 2/3 fever) reported during the 7-day follow-up period after vaccination, including all RSV formulations.

For any Grade 2/3 general AEs and any related SAEs, the incidence rate estimate ($\hat{IR}$) has been transformed in a [0,1] desirability index using the following function:

where $\hat{IR}$ is the incidence rate estimated by the model. This function allocated a desirability value of 1, 0.5 and 0 to incidence rate equal to 0.1, 0.25 and 0.5 respectively (see Figure ).

**Figure S1 Desirability function for the incidence rate of Grade 2/3 general AEs and related SAEs for each investigational RSV vaccine formulation**


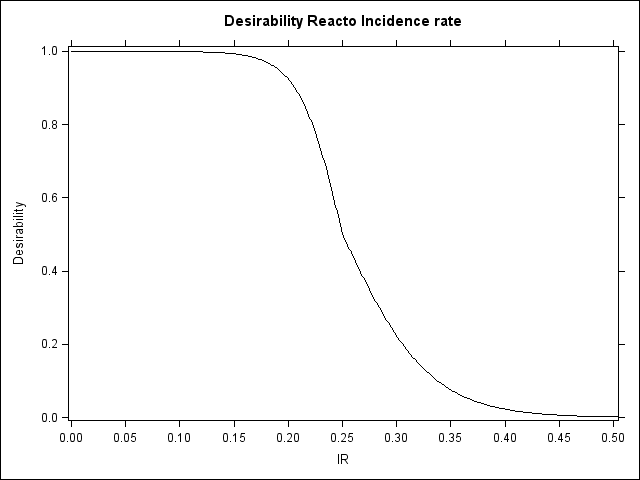


For Grade 2/3 fever, the incidence rate estimate ($\hat{IR}$) was transformed in a [0,1] desirability index using the following function:

where $\hat{IR}$ is the incidence rate estimated by the model. As illustrated in Figure , the function allocated desirability values of 1, 0.5 and 0 to incidence rate equal to 0, 0.05 and 0.1 respectively.

**Figure S2 Desirability function for the incidence rate of Grade 2/3 fever for each investigational RSV vaccine formulation**


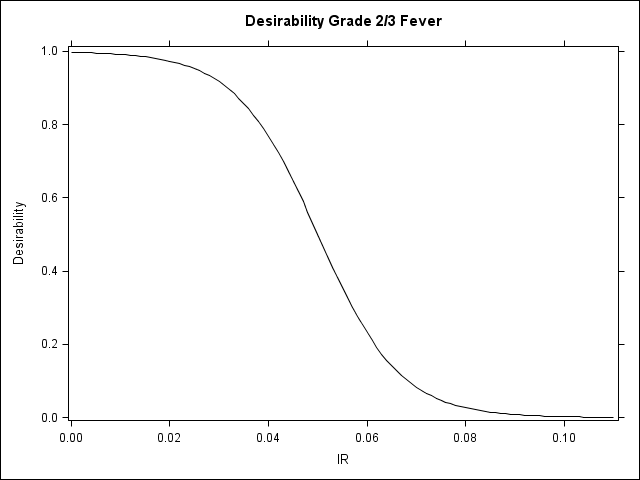


Finally, the reactogenicity index has been computed by taking the geometric mean of the 2 indices:

**Immunogenicity**

A response-surface model has been fitted on the log-transformed titre for each immune response (neutralising anti-RSV-A and PCA), including all RSV formulations.

As formulations inducing a high immune response were considered suitable, the lower limit (LL) of the estimated GMT/C adjusted for pre-vaccination titres was the statistical criterion considered for decision making.

*Neutralising anti-RSV-A titres*

The LL of the GMT estimate was transformed into a [0,1] desirability index using the function:

where LL is the lower limit of the 95% confidence interval of the GMT adjusted for pre-vaccination titers in log base 2. The function was chosen to have a desirability of 0 at LL value ≤ 6 log2 (=128), and a desirability of 1 at LL value ≥ 13 log2. This function is illustrated in Figure .

**Figure S3 Desirability function for neutralising anti-RSV-A GMTs for each investigational RSV vaccine formulation**


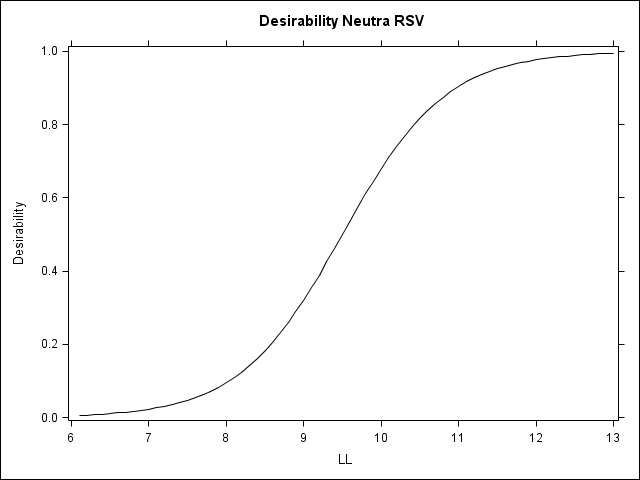


*PCA concentrations*

The LL of the GMC adjusted for pre-vaccination titers estimate has been transformed using the following function:

As illustrated in Figure , a PCA response of 25, 150 and 400 µg/mL had a desirability value of 0, 0.5 and 1 respectively.

**Figure S4 Desirability function for PCA concentrations for each investigational RSV vaccine formulation**


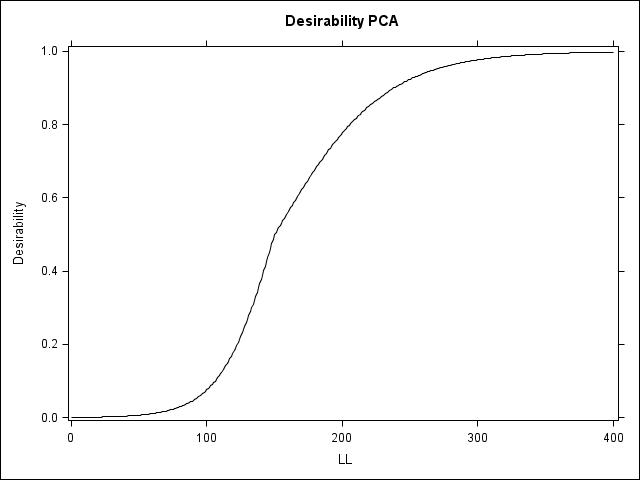


Finally, the immunogenicity index was computed by taking the geometric mean of the 2 indices:

**Overall desirability index**

The overall desirability index has been obtained by computing the following weighted geometric mean: D = DR^0.4^ * DI^0.6^

Finally, to address the robustness of the ranking, an evaluation of the uncertainty was performed using a bootstrap approach.

**Supplementary data 3.** Safety assessments

Solicited local (pain, redness, and swelling at injection site) and general (fatigue, fever, gastrointestinal symptoms, and headache) AEs were recorded for 7 days, and unsolicited AEs for 30 days post-vaccination, using diary cards.

All AEs were graded on a scale of 1 (mild) to 3 (severe). All solicited local AEs were considered as related to vaccination. The clinical investigators assessed relatedness of all other AEs. Nasal swabs were collected from women who had a medically-attended respiratory tract infection.

Hematological (hemoglobin, white blood cell, lymphocyte, neutrophil, and eosinophil levels, and platelet counts) and biochemical (alanine amino-transferase [ALT], aspartate amino-transferase [AST], and creatinine) parameters were measured on Days 0, 7, 30, 60, and 90.

**Supplementary data 4.** Immunogenicity assessments

RSV neutralization assays were performed by incubating fixed amounts of RSV-A or RSV-B with serial dilutions of test serum. The serum-virus mixture was transferred onto a Vero cell monolayer and incubated for 3 days. RSV-infected cells were then detected using a primary antibody directed against RSV and a secondary antibody conjugated with fluorescein isothiocyanate. For each serum dilution, the reduction in the number of plaques versus control wells was calculated. Serum neutralizing antibody titers, expressed as Estimated Dilution 60, correspond to the inverse of the interpolated serum dilution yielding a 60% reduction in the number of plaques compared to control wells.

The PCA assay was based on competitive binding of labelled antibody (Palivizumab-Horseradish Peroxidase) versus non-labelled antibody (Palivizumab-like antibodies in serum) targeting the same epitope on the PreF vaccine antigen using enzyme-linked immunosorbent assay (ELISA). Antibody concentrations were expressed as Palivizumab-equivalent antibodies in microgram per milliliter (µg/mL).

RSV-specific total immunoglobulin (IgG) antibody titers and IgG1 antibody titers were measured up to Day 30 using enzyme-linked immunosorbent assays (ELISA).

**Supplementary data 5.** Statistical analyses

Safety analyses were performed on the Exposed Set, including all women with documented vaccination. Immunogenicity analyses were performed on the Per-Protocol Set, including all women meeting eligibility criteria, who received the study vaccine according to protocol procedures, complied with the blood sampling schedule, and for whom immunogenicity results were available for ≥1 assay at the corresponding timepoint.

Antibody geometric mean titers (GMTs)/geometric mean concentrations (GMCs) and seropositivity rates were calculated with 95% confidence intervals (CIs). For results below the cut-off, an arbitrary value of half of the cut-off was considered for calculation of GMC and fold increase. Exploratory evaluations compared neutralizing antibody GMTs against RSV-A and RSV-B, and PCA GMCs between RSV-PreF vaccine groups on Day 30, using an analysis of covariance model with vaccine group as fixed effect and pre-vaccination titer/concentration as covariate. Pairwise comparisons were made using the Tukey multiple comparison adjustment. Similar exploratory analyses were performed for RSV-F-specific total IgG and IgG1 antibody titers at Day 30 in a random subset of 50 participants per group.

Percentages of women with solicited and unsolicited AEs were tabulated with 95% CIs up to Day 7 or Day 30. Descriptive analyses on percentages of women with any grade 2/3 general AE, grade 2/3 fever, and/or vaccine-related SAE within 7 days post-vaccination were performed by group.

All analyses were performed using SAS Drug Development software (version 4.3.4).

**Supplementary Table 1.** Desirability indices based on reactogenicity and safety (Exposed Set up to Day 7) and immunogenicity (Per-Protocol Set up to Day 30).

|  | **Reactogenicity index** | | | **Immunogenicity index** | | | **Overall desirability index** |
| --- | --- | --- | --- | --- | --- | --- | --- |
| **Group** | **DR1** | **DR2** | **DR** | **DI1** | **DI2** | **DI** | **DR^0.4^ * DI^0.6^** |
| 120 RSV-PreF | 0.98 | 0.99 | 0.98 | 0.68 | 0.02 | 0.12 | 0.27 |
| 60 RSV-PreF | 0.55 | 0.99 | 0.74 | 0.66 | 0.02 | 0.11 | 0.24 |
| 30 RSV-PreF | 0.48 | 1.00 | 0.69 | 0.56 | 0.01 | 0.08 | 0.19 |
| Control | 0.75 | 1.00 | 0.86 | 0.09 | 0.00 | 0.01 | 0.05 |

30 RSV-PreF/60 RSV-PreF/120 RSV-PreF, group of women who received 1 dose of the unadjuvanted respiratory syncytial virus (RSV) vaccine containing 30/60/120μg of RSV pre-fusion F protein (PreF); Control, group of women who received 1 dose of the placebo; DR, reactogenicity index; DR1, desirability index for any grade 2/3 general adverse events or any related serious adverse events; DR2, desirability index for grade 2/3 fever; DI, immunogenicity index; DI1, desirability index for neutralizing anti RSV-A; DI2, desirability index for palivizumab-competing antibody concentrations. Note: As there are only two participants for the reactogenicity endpoint (grade 2/3 fever), a logistic regression model could not be fitted. Thus, point estimates were used from sample to estimate the incidence rate for grade 2/3 fever.

**Supplementary Table 2**. Exploratory comparisons between groups in terms of the percentage of women who reported any grade 2/3 AE, fever >38.5°C or vaccine-related SAE during the 7-day post-vaccination period (Exposed Set).

| **Group 1** | | | |  | **Group 2** | | | |  | **Difference in percentages (Group 1 minus Group 2)** |
| --- | --- | --- | --- | --- | --- | --- | --- | --- | --- | --- |
|  | **N** | **n** | **%** |  |  | N | **n** | **%** |  | **% (95% CI)** |
| 30 RSV-PreF | 100 | 34 | 34.0 |  | Control | 102 | 28 | 27.5 |  | 6.55 (-6.21, 19.17) |
| 60 RSV-PreF | 99 | 35 | 35.4 |  | Control | 102 | 28 | 27.5 |  | 7.90 (-4.96, 20.59) |
| 120 RSV-PreF | 99 | 28 | 28.3 |  | Control | 102 | 28 | 27.5 |  | 0.83 (-11.57, 13.27) |
| 60 RSV-PreF | 99 | 35 | 35.4 |  | 30 RSV-PreF | 100 | 34 | 34.0 |  | 1.35 (-11.84, 14.52) |
| 120 RSV-PreF | 99 | 28 | 28.3 |  | 30 RSV-PreF | 100 | 34 | 34.0 |  | -5.72 (-18.46, 7.19) |
| 120 RSV-PreF | 99 | 28 | 28.3 |  | 60 RSV-PreF | 99 | 35 | 35.4 |  | -7.07 (-19.89, 5.95) |

30 RSV-PreF/60 RSV-PreF/120 RSV-PreF, group of women who received 1 dose of the unadjuvanted respiratory syncytial virus (RSV) vaccine containing 30/60/120μg of RSV pre-fusion F protein (PreF); Control, group of women who received 1 dose of the placebo; N, total number of participants; n (%), number (percentage) of participants in a given category; (S)AE, (serious) adverse event; 95% CI, 95% confidence interval.
